# Supplementary material for: The Impact of COVID-19 and the Pandemic on Tinnitus: A Systematic Review
Source: J Clin Med. 2021 Jun 23;10(13):2763. doi: 10.3390/jcm10132763 (PMC8268057; doi:10.3390/jcm10132763)
Supplement: Supplementary file 1 [file jcm-10-02763-s001.zip › jcm-1267175-supplementary.pdf]

Table S1. Preferred Reporting Items for Systematic reviews and Meta-analyses (PRISMA) checklist

Section and Topic	Item #	Checklist item	Location where item is reported
**TITLE**			
Title	1	Identify the report as a systematic review.	L2
**ABSTRACT**			
Abstract	2	See the PRISMA 2020 for Abstracts checklist.	L11-23
**INTRODUCTION**			
Rationale	3	Describe the rationale for the review in the context of existing knowledge.	L64-67
Objectives	4	Provide an explicit statement of the objective(s) or question(s) the review addresses.	L70-73
**METHODS**			
Eligibility criteria	5	Specify the inclusion and exclusion criteria for the review and how studies were grouped for the syntheses.	L82
Information sources	6	Specify all databases, registers, websites, organisations, reference lists and other sources searched or consulted to identify studies. Specify the date when each source was last searched or consulted.	L99-103
Search strategy	7	Present the full search strategies for all databases, registers and websites, including any filters and limits used.	L107-111
Selection process	8	Specify the methods used to decide whether a study met the inclusion criteria of the review, including how many reviewers screened each record and each report retrieved, whether they worked independently, and if applicable, details of automation tools used in the process.	L120-123
Data collection process	9	Specify the methods used to collect data from reports, including how many reviewers collected data from each report, whether they worked independently, any processes for obtaining or confirming data from study investigators, and if applicable, details of automation tools used in the process.	L125-133
Data items	10a	List and define all outcomes for which data were sought. Specify whether all results that were compatible with each outcome domain in each study were sought (e.g. for all measures, time points, analyses), and if not, the methods used to decide which results to collect.	L128-132
	10b	List and define all other variables for which data were sought (e.g. participant and intervention characteristics, funding sources). Describe any assumptions made about any missing or unclear information.	L141
Study risk of bias assessment	11	Specify the methods used to assess risk of bias in the included studies, including details of the tool(s) used, how many reviewers assessed each study and whether they worked independently, and if applicable, details of automation tools used in the process.	L13-144
Effect measures	12	Specify for each outcome the effect measure(s) (e.g. risk ratio, mean difference) used in the synthesis or presentation of results.	L149-147
Synthesis methods	13a	Describe the processes used to decide which studies were eligible for each synthesis (e.g. tabulating the study intervention characteristics and comparing against the planned groups for each synthesis (item #5)).	L153
	13b	Describe any methods required to prepare the data for presentation or synthesis, such as handling of missing summary statistics, or data conversions.	155
	13c	Describe any methods used to tabulate or visually display results of individual studies and syntheses.	L157
	13d	Describe any methods used to synthesize results and provide a rationale for the choice(s). If meta-analysis was performed, describe the model(s), method(s) to identify the presence and extent of statistical heterogeneity, and software package(s) used.	L155
	13e	Describe any methods used to explore possible causes of heterogeneity among study results (e.g. subgroup analysis, meta-regression).	L160
	13f	Describe any sensitivity analyses conducted to assess robustness of the synthesized results.	L150
Reporting bias assessment	14	Describe any methods used to assess risk of bias due to missing results in a synthesis (arising from reporting biases).	L136
Certainty assessment	15	Describe any methods used to assess certainty (or confidence) in the body of evidence for an outcome.	L144
**RESULTS**			
Study selection	16a	Describe the results of the search and selection process, from the number of records identified in the search to the number of studies included in the review, ideally using a flow diagram.	Fig 1
	16b	Cite studies that might appear to meet the inclusion criteria, but which were excluded, and explain why they were excluded.	Fig 1
Study characteristics	17	Cite each included study and present its characteristics.	Table 2-4
Risk of bias in studies	18	Present assessments of risk of bias for each included study.	L191-198 and Tables 2-4
Results of individual	19	For all outcomes, present, for each study: (a) summary statistics for each group (where appropriate) and (b) an effect estimate and its precision (e.g. confidence/credible interval),	Fig 2

studies		ideally using structured tables or plots.	
Results of syntheses	20a	For each synthesis, briefly summarise the characteristics and risk of bias among contributing studies.	L379
	20b	Present results of all statistical syntheses conducted. If meta-analysis was done, present for each the summary estimate and its precision (e.g. confidence/credible interval) and measures of statistical heterogeneity. If comparing groups, describe the direction of the effect.	Figure 2
	20c	Present results of all investigations of possible causes of heterogeneity among study results.	L376-382
	20d	Present results of all sensitivity analyses conducted to assess the robustness of the synthesized results.	L377
Reporting biases	21	Present assessments of risk of bias due to missing results (arising from reporting biases) for each synthesis assessed.	L378
Certainty of evidence	22	Present assessments of certainty (or confidence) in the body of evidence for each outcome assessed.	L383
**DISCUSSION**			
Discussion	23a	Provide a general interpretation of the results in the context of other evidence.	L681
	23b	Discuss any limitations of the evidence included in the review.	L686-700
	23c	Discuss any limitations of the review processes used.	L689
	23d	Discuss implications of the results for practice, policy, and future research.	L700-738
**OTHER INFORMATION**			
Registration and protocol	24a	Provide registration information for the review, including register name and registration number, or state that the review was not registered.	L76
	24b	Indicate where the review protocol can be accessed, or state that a protocol was not prepared.	L77
	24c	Describe and explain any amendments to information provided at registration or in the protocol.	L80
Support	25	Describe sources of financial or non-financial support for the review, and the role of the funders or sponsors in the review.	Funding statement
Competing interests	26	Declare any competing interests of review authors.	Conflicts of interest statement
Availability of data, code and other materials	27	Report which of the following are publicly available and where they can be found: template data collection forms; data extracted from included studies; data used for all analyses; analytic code; any other materials used in the review.	Appendix A4

Table S2. Search strategies

Search strategy for **PubMed (MEDLINE)** database ran on 28 March 2021.

Category	Number	Search Strategy	No. of records returned
Condition	S1 tinnitus	tinnitus*	14,087
Disease	S2	COVID-19* OR coronavirus	127,061
Limiters		Human<br>Search modes: Boolean/phrase	
Combined S1 and S2			29 records

Records identified via Pubmed

1: Chirakkal P, Al Hail AN, Zada N, Vijayakumar DS. COVID-19 and Tinnitus. Ear Nose Throat J. 2021 Apr;100(2_suppl):160S-162S. doi: 10.1177/0145561320974849. Epub 2020 Dec 4. PMID: 33275033; PMCID: PMC7720028.
2: Viola P, Ralli M, Pisani D, Malanga D, Sculco D, Messina L, Laria C, Aragona T, Leopardi G, Ursini F, Scarpa A, Topazio D, Cama A, Vespertini V, Quintieri F, Cosco L, Cunsolo EM, Chiarella G. Tinnitus and equilibrium disorders in COVID-19 patients: preliminary results. Eur Arch Otorhinolaryngol. 2020 Oct 23:1–6. doi: 10.1007/s00405-020-06440-7. Epub ahead of print. PMID: 33095432; PMCID: PMC7582442.
3: Almufarrij I, Uus K, Munro KJ. Does coronavirus affect the audio-vestibular system? A rapid systematic review. Int J Audiol. 2020 Jul;59(7):487-491. doi: 10.1080/14992027.2020.1776406. Epub 2020 Jun 12. PMID: 32530326.
4: Prayuenyong P, Kasbekar AV, Baguley DM. Clinical Implications of Chloroquine and Hydroxychloroquine Ototoxicity for COVID-19 Treatment: A Mini-Review. Front Public Health. 2020 May 29;8:252. doi: 10.3389/fpubh.2020.00252. PMID: 32574312; PMCID: PMC7273970.
5: Freni F, Meduri A, Gazia F, Nicastro V, Galletti C, Aragona P, Galletti C, Galletti B, Galletti F. Symptomatology in head and neck district in coronavirus disease (COVID-19): A possible neuroinvasive action of SARS-CoV-2. Am J Otolaryngol. 2020 Sep-Oct;41(5):102612. doi: 10.1016/j.amjoto.2020.102612. Epub 2020 Jun 18. PMID: 32574896; PMCID: PMC7301823.
6: Elibol E. Otolaryngological symptoms in COVID-19. Eur Arch Otorhinolaryngol. 2021 Apr;278(4):1233-1236. doi: 10.1007/s00405-020-06319-7. Epub 2020 Sep 1. PMID: 32875391; PMCID: PMC7461752.
7: Maharaj S, Bello Alvarez M, Mungul S, Hari K. Otologic dysfunction in patients with COVID-19: A systematic review. Laryngoscope Investig Otolaryngol. 2020 Nov 17;5(6):1192-1196. doi: 10.1002/lio2.498. PMID: 33365394; PMCID: PMC7752038.
8: Cui C, Yao Q, Zhang D, Zhao Y, Zhang K, Nisenbaum E, Cao P, Zhao K, Huang X, Leng D, Liu C, Li N, Luo Y, Chen B, Casiano R, Weed D, Sargi Z, Telischi F, Lu H, Denneny JC 3rd, Shu Y, Liu X. Approaching Otolaryngology Patients During the COVID-19 Pandemic. Otolaryngol Head Neck Surg. 2020 Jul;163(1):121-131. doi: 10.1177/0194599820926144. Epub 2020 May 12. PMID: 32396445; PMCID: PMC7218357
9: Munro KJ, Uus K, Almufarrij I, Chaudhuri N, Yioe V. Persistent self-reported changes in hearing and tinnitus in post-hospitalisation COVID-19 cases. Int J Audiol. 2020 Dec;59(12):889-890. doi: 10.1080/14992027.2020.1798519. Epub 2020 Jul 31. PMID: 32735466.
10: Beukes EW, Baguley DM, Jacquemin L, Lourenco MPCG, Allen PM, Onozuka J, Stockdale D, Kaldo V, Andersson G, Manchaiah V. Changes in Tinnitus Experiences During the COVID-19 Pandemic. Front Public Health. 2020 Nov 5;8:592878. doi: 10.3389/fpubh.2020.592878. PMID: 33251179; PMCID: PMC7676491.
11: Anzivino R, Sciancalepore PI, Petrone P, D'Elia A, Petrone D, Quaranta N. Tinnitus revival during COVID-19 lockdown: how to deal with it? Eur Arch Otorhinolaryngol. 2021 Jan;278(1):295-296. doi: 10.1007/s00405-020-06147-9. Epub 2020 Jun 22. PMID: 32572563; PMCID: PMC7307941.
12: Özçelik Korkmaz M, Eğilmez OK, Özçelik MA, Güven M. Otolaryngological manifestations of hospitalised patients with confirmed COVID-19 infection. Eur Arch Otorhinolaryngol. 2020 Oct 3:1–11. doi: 10.1007/s00405-020-06396-8. Epub ahead of print. PMID: 33011957; PMCID: PMC7532931.
13: Saunders GH, Roughley A. Audiology in the time of COVID-19: practices and opinions of audiologists in the UK. Int J Audiol. 2020 Sep 10:1-8. doi: 10.1080/14992027.2020.1814432. Epub ahead of print. PMID: 32909474.

14: Narozny W, Tretiakow D, Skorek A. Tinnitus in COVID-19 Pandemic. Ear Nose Throat J. 2021 Jan 20:145561320988364. doi: 10.1177/0145561320988364. Epub ahead of print. PMID: 33470830.
15: Liang Y, Xu J, Chu M, Mai J, Lai N, Tang W, Yang T, Zhang S, Guan C, Zhong F, Yang L, Liao G. Neurosensory dysfunction: A diagnostic marker of early COVID-19. Int J Infect Dis. 2020 Sep;98:347-352. doi: 10.1016/j.ijid.2020.06.086. Epub 2020 Jun 29. PMID: 32615326; PMCID: PMC7323654.
16: Maharaj S, Hari K. Congenital Inner Ear Abnormalities and COVID-19-Related Ear Infections. Ear Nose Throat J. 2020 Oct 23:145561320968934. doi: 10.1177/0145561320968934. Epub ahead of print. PMID: 33095653.
17: Naylor G, Burke LA, Holman JA. Covid-19 Lockdown Affects Hearing Disability and Handicap in Diverse Ways: A Rapid Online Survey Study. Ear Hear. 2020 Nov/Dec;41(6):1442-1449. doi: 10.1097/AUD.0000000000000948. PMID: 33136621.
18: Lamounier P, Franco Gonçalves V, Ramos HVL, Gobbo DA, Teixeira RP, Dos Reis PC, Bahmad F Jr, Cândido Costa C. A 67-Year-Old Woman with Sudden Hearing Loss Associated with SARS-CoV-2 Infection. Am J Case Rep. 2020 Nov 3;21:e927519. doi: 10.12659/AJCR.927519. PMID: 33139689; PMCID: PMC7650213.
19: Little C, Cosetti MK. A Narrative Review of Pharmacologic Treatments for COVID-19: Safety Considerations and Ototoxicity. Laryngoscope. 2021 Jan 24. doi: 10.1002/lary.29424. Epub ahead of print. PMID: 33491234.
20: Aazh H, Swanepoel W, Moore BCJ. Telehealth tinnitus therapy during the COVID-19 outbreak in the UK: uptake and related factors. Int J Audiol. 2020 Oct 1:1-6. doi: 10.1080/14992027.2020.1822553. Epub ahead of print. PMID: 33000663.
21: Cappy P, Candotti D, Sauvage V, Lucas Q, Boizeau L, Gomez J, Enouf V, Chabli L, Pillonel J, Tiberghien P, Morel P, Laperche S. No evidence of SARS-CoV-2 transfusion transmission despite RNA detection in blood donors showing symptoms after donation. Blood. 2020 Oct 15;136(16):1888-1891. doi: 10.1182/blood.2020008230. PMID: 32871595; PMCID: PMC7568032.
22: Schlee W, Hølleland S, Bulla J, Simoes J, Neff P, Schoisswohl S, Woelflick S, Schecklmann M, Schiller A, Staudinger S, Probst T, Langguth B. The Effect of Environmental Stressors on Tinnitus: A Prospective Longitudinal Study on the Impact of the COVID-19 Pandemic. J Clin Med. 2020 Aug 26;9(9):2756. doi: 10.3390/jcm9092756. PMID: 32858835; PMCID: PMC7565885.
23: Vijayasundaram S, Karthikeyan P, Mehta SD. Proficiency of virtual follow-up amongst tinnitus patients who underwent intratympanic steroid therapy amidst COVID 19 pandemic. Am J Otolaryngol. 2020 Nov-Dec;41(6):102680. doi: 10.1016/j.amjoto.2020.102680. Epub 2020 Aug 15. PMID: 32861124; PMCID: PMC7428771.
24: Micarelli A, Granito I, Carlino P, Micarelli B, Alessandrini M. Self-perceived general and ear-nose-throat symptoms related to the COVID-19 outbreak: a survey study during quarantine in Italy. J Int Med Res. 2020 Oct;48(10):300060520961276. doi: 10.1177/0300060520961276. PMID: 33081538.
25: Xia L, He G, Feng Y, Yu X, Zhao X, Yin S, Chen Z, Wang J, Fan J, Dong C. COVID-19 associated anxiety enhances tinnitus. PLoS One. 2021 Feb 5;16(2):e0246328. doi: 10.1371/journal.pone.0246328. PMID: 33544744; PMCID: PMC7864409.
26: Saniasiaya J, Kulasegarah J. Auditory Cinchonism in COVID Era. Ear Nose Throat J. 2020 Nov;99(9):597-598. doi: 10.1177/0145561320947255. Epub 2020 Aug 3. PMID: 32744901.
27: Kunin A, Sargheini N, Birkenbihl C, Moiseeva N, Fröhlich H, Golubnitschaja O. Voice perturbations under the stress overload in young individuals: phenotyping and suboptimal health as predictors for cascading pathologies. EPMA J. 2020 Nov 12;11(4):1-11. doi: 10.1007/s13167-020-00229-8. Epub ahead of print. PMID: 33200009; PMCID: PMC7658305.
28: Gallus R, Melis A, Rizzo D, Piras A, De Luca LM, Tramaloni P, Serra A, Longoni E, Soro GM, Bussu F. Audiovestibular symptoms and sequelae in COVID-19 patients. J Vestib Res. 2021 Feb 10. doi: 10.3233/VES-201505. Epub ahead of print. PMID: 33579886.
29: Almufarrij I, Munro KJ. One year on: an updated systematic review of SARS-CoV-2, COVID-19 and audio-vestibular symptoms. Int J Audiol. 2021 Mar 22:1-11. doi: 10.1080/14992027.2021.1896793. Epub ahead of print. PMID: 33750252.

Search strategy for **CINAHL Complete and Academic Search Complete** databases run on 28 March 2021

Category	Number	Search Strategy	No. of records returned
Condition	S1 tinnitus	tinnitus*	8,396
Disease	S2	COVID-19* OR coronavirus	100,185
Limiters		Human<br>Search modes: Boolean/phrase	
Combined S1 and S2			21 records (23 but duplicates automatically removed)

Records Identified from CINAHL Complete and Academic Search Complete.

1. Lamounier, P., Gonçalves, V. F., Ramos, H. V. L., Gobbo, D. A., Teixeira, R. P., dos Reis, P. C., Bahmad Jr., F., & Costa, C. C. (2020). A 67-Year-Old Woman with Sudden Hearing Loss Associated with SARS-CoV-2 Infection. *American Journal of Case Reports*, *21*, 1–6. https://doi-org.libproxy.lamar.edu/10.12659/AJCR.927519
2. Cui, C., Yao, Q., Zhang, D., Zhao, Y., Zhang, K., Nisenbaum, E., Cao, P., Zhao, K., Huang, X., Leng, D., Liu, C., Li, N., Luo, Y., Chen, B., Casiano, R., Weed, D., Sargi, Z., Telischi, F., Lu, H., & Denneny III, J. C. (2020). Approaching Otolaryngology Patients During the COVID-19 Pandemic. *Otolaryngology-Head & Neck Surgery*, *163*(1), 121–131. https://doi-org.libproxy.lamar.edu/10.1177/0194599820926144
3. Saniasiaya, J., & Kulasegarah, J. (2020). Auditory Cinchonism in COVID Era. *ENT: Ear, Nose & Throat Journal*, *99*(9), 597–598. https://doi-org.libproxy.lamar.edu/10.1177/0145561320947255
4. Chirakkal, P., Al Hail, A. N., Zada, N., & Vijayakumar, D. S. (2021). COVID-19 and Tinnitus. *ENT: Ear, Nose & Throat Journal*, *100*, 160S–162S. https://doi-org.libproxy.lamar.edu/10.1177/0145561320974849
5. Xia, L., He, G., Feng, Y., Yu, X., Zhao, X., Yin, S., Chen, Z., Wang, J., Fan, J., & Dong, C. (2021). COVID-19 associated anxiety enhances tinnitus. *PLoS ONE*, *16*(2), 1–22. https://doi-org.libproxy.lamar.edu/10.1371/journal.pone.0246328
6. COVID-19 May Exacerbate Tinnitus Symptoms. (2021). *ASHA Leader*, *26*(1), 21.
7. Saniasiaya, J., & Kulasegarah, J. (2021). Dizziness and COVID-19. *ENT: Ear, Nose & Throat Journal*, *100*(1), 29–30. https://doi-org.libproxy.lamar.edu/10.1177/0145561320959573
8. Almufarrij, I., Uus, K., & Munro, K. J. (2020). Does coronavirus affect the audio-vestibular system? A rapid systematic review. *International Journal of Audiology*, *59*(7), 487–491. https://doi-org.libproxy.lamar.edu/10.1080/14992027.2020.1776406
9. Hobin, T. (2020). Hearing Protection Field Test 2020 – Some of the Best Gear For Your Ears: A musician should protect their hearing as if their career depended on it. *International Musician*, *118*(5), 13.
10. Liang, Y., Xu, J., Chu, M., Mai, J., Lai, N., Tang, W., Yang, T., Zhang, S., Guan, C., Zhong, F., Yang, L., & Liao, G. (2020). Neurosensory dysfunction: A diagnostic marker of early COVID-19. *International Journal of Infectious Diseases*, *98*, 347–352. https://doi-org.libproxy.lamar.edu/10.1016/j.ijid.2020.06.086
11. Almufarrij, I., & Munro, K. J. (2021). One year on: an updated systematic review of SARS-CoV-2, COVID-19 and audio-vestibular symptoms. *International Journal of Audiology*, 1–11. https://doi-org.libproxy.lamar.edu/10.1080/14992027.2021.1896793
12. Özçelik Korkmaz, M., Eğilmez, O. K., Özçelik, M. A., & Güven, M. (2020). Otolaryngological manifestations of hospitalised patients with confirmed COVID-19 infection. *European Archives of Oto-Rhino-Laryngology*, 1–11. https://doi-org.libproxy.lamar.edu/10.1007/s00405-020-06396-8
13. Elibol, E. (2021). Otolaryngological symptoms in COVID-19. *European Archives of Oto-Rhino-Laryngology*, *278*(4), 1233–1236. https://doi-org.libproxy.lamar.edu/10.1007/s00405-020-06319-7
14. Maharaj, S., Bello Alvarez, M., Mungul, S., & Hari, K. (2020). Otologic dysfunction in patients with COVID-19: A systematic review. *Laryngoscope Investigative Otolaryngology*, *5*(6), 1192–1196. https://doi-org.libproxy.lamar.edu/10.1002/lio2.498
15. Beata Skarzynska, M., Krol, B., Czajka, N., & Czajka, L. (2020). Ototoxicity of Drugs Used in the Treatment of Covid-19. *Journal of Hearing Science*, *10*, 9–20. https://doi-org.libproxy.lamar.edu/10.17430/JHS.2020.10.1.1
16. Munro, K. J., Uus, K., Almufarrij, I., Chaudhuri, N., & Yioe, V. (2020). Persistent self-reported changes in hearing and tinnitus in post-hospitalisation COVID-19 cases. *International Journal of Audiology*, *59*(12), 889–890. https://doi-org.libproxy.lamar.edu/10.1080/14992027.2020.1798519
17. Zitelli, L., & Mormer, E. (2020). Smartphones and Hearing Loss: There's an App for That! *Seminars in Hearing*, *41*(4), 266–276. https://doi-org.libproxy.lamar.edu/10.1055/s-0040-1718712
18. Schlee, W., Hølleland, S., Bulla, J., Simoes, J., Neff, P., Schoisswohl, S., Woelflick, S., Schecklmann, M., Schiller, A., Staudinger, S., Probst, T., & Langguth, B. (2020). The Effect of Environmental Stressors on Tinnitus: A Prospective Longitudinal Study on the Impact of the COVID-19 Pandemic. *Journal of Clinical Medicine*, *9*(9), 2756. https://doi-org.libproxy.lamar.edu/10.3390/jcm9092756
19. Viola, P., Ralli, M., Pisani, D., Malanga, D., Sculco, D., Messina, L., Laria, C., Aragona, T., Leopardi, G., Ursini, F., Scarpa, A., Topazio, D., Cama, A., Vespertini, V., Quintieri, F., Cosco, L., Cunsolo, E. M., & Chiarella, G. (2020). Tinnitus and equilibrium disorders in COVID-19 patients: preliminary results. *European Archives of Oto-Rhino-Laryngology*, 1–6. https://doi-org.libproxy.lamar.edu/10.1007/s00405-020-06440-7
20. Anzivino, R., Sciancalepore, P. I., Petrone, P., D'Elia, A., Petrone, D., & Quaranta, N. (2021). Tinnitus revival during COVID-19 lockdown: how to deal with it? *European Archives of Oto-Rhino-Laryngology*, *278*(1), 295–296. https://doi-org.libproxy.lamar.edu/10.1007/s00405-020-06147-9
21. СОЛОВЬЕВ, А. И., УСКОВ, А. Н., & МОСКАЛЕВ, А. В. (2020). Химиопрофилактика Малярии При Длительном Пребывании На Эндемичной Территории: Опыт Применения Мефлохина И Хлорохина.

Epidemiologiia i Infektsionnye Bolezni - Aktualnye Voprosy, *10*(2), 84–89. https://doi-org.libproxy.lamar.edu/10.18565/epidem.2020.10.2.84–9

Search strategy for **Web of Science** database run on 28 March 2021.

Category	Number	Search Strategy	No. of records returned
Condition	S1 tinnitus	tinnitus*	9,464
Disease	S2	COVID-19* OR coronavirus	115, 737
Limiters		Human<br>Search modes: Boolean/phrase	
Combined S1 and S2			25 records

Records identified from Web of Science.

1. Cui, C., Yao, Q., Zhang, D., Zhao, Zhang, K., Nisenbaum, E., Cao, P. Y., Zhao, K.Q., Huang, X. L., Leng, D. W., Liu, C. H., Li, N., Luo, Y., Chen, B., Roy, C., Weed, D., Sargi, Z., Telischi, F., Lu, H. Z., Denneny, J.C., Shu, Y. L., Liu, X. Z. (2020). Approaching Otolaryngology Patients During the COVID-19 Pandemic. OTOLARYNGOLOGY-HEAD AND NECK SURGERY. DOI 10.1177/0194599820926144 PM 323964
2. Freni, F., Meduri, A., Gazia, F., Nicastro, V., Galletti, C., Aragona, P., Galletti, B., Galletti, F. (2020). Symptomatology in head and neck district in coronavirus disease (COVID-19): A possible neuroinvasive action of SARS-CoV-2. AMERICAN JOURNAL OF OTOLARYNGOLOGY. DOI 10.1016/j.amjoto.2020.102612. PM 3257489
3. Liang, Y. J., Xu, J.B., Chu, M., Mai, J.B., Lai, N. M., Tang, W., Yang, T. J., Zhang, S. E., Guan, C. Y., Zhong, F., Yang, L. P., Liao, G. Q. (2020). Neurosensory dysfunction: A diagnostic marker of early COVID-19. INTERNATIONAL JOURNAL OF INFECTIOUS DISEASES. DOI 10.1016/j.ijid.2020.06.086. PM 32615326.
4. Prayuenyong, P. Kasbekar, A. V., Baguley, D. M., (2020). Clinical Implications of Chloroquine and Hydroxychloroquine Ototoxicity for COVID-19 Treatment: A Mini-Review. FRONTIERS IN PUBLIC HEALTH. DOI 10.3389/fpubh.2020.00252. PM 32574312.
5. Munro, K.J., Uus, K., Almufarrij, I., Chaudhuri, N., Yioe, V. (2020). Persistent self-reported changes in hearing and tinnitus in post-hospitalisation COVID-19 case. INTERNATIONAL JOURNAL OF AUDIOLOGY. DOI 10.1080/14992027.2020.1798519. PM 32735466.
6. Almufarrij, I. Uus, K., Munro, K. J. (2020). Does coronavirus affect the audio-vestibular system? A rapid systematic review. INTERNATIONAL JOURNAL OF AUDIOLOGY. DOI 10.1080/14992027.2020.1776406. PM 32530326.
7. Korkmaz, M. O., Egilmez, O. K., Ozcelik, M. A., Guven, M. (2020). Otolaryngological manifestations of hospitalised patients with confirmed COVID-19 infection. EUROPEAN ARCHIVES OF OTO-RHINO-LARYNGOLOGY. DOI 10.1007/s00405-020-06396-8. PM 33011957.
8. Elibol, E. Otolaryngological symptoms in COVID-19. (2021). EUROPEAN ARCHIVES OF OTO-RHINO-LARYNGOLOGY. DOI 10.1007/s00405-020-06319-7. PM 32875391
9. Chirakkal, P. Al Hail, A. N., Zada, N., Vijayakumar, D. S. (2021). COVID-19 and Tinnitus. ENT-EAR NOSE & THROAT JOURNAL. DOI 10.1177/0145561320974849. PM 33275033.
10. Kunin, A., Sargheini, N., Birkenbihl, C., Moiseeva, N., Frohlich, H., Golubnitschaja, O. (2020). Voice perturbations under the stress overload in young individuals: phenotyping and suboptimal health as predictors for cascading pathologies. EPMA JOURNAL. DOI 10.1007/s13167-020-00229-8. PM 33200009.
11. Lamounier, P., Goncalves, V. F., Ramos, H. V. L., Gobbo, D. A., Teixeira, R. P., dos Reis, P. C., Bahmad, F., Costa, C. C. (2020). A 67-Year-Old Woman with Sudden Hearing Loss Associated with SARS-CoV-2 Infection. AMERICAN JOURNAL OF CASE REPORTS. DOI 10.12659/AJCR.927519. PM 33139689.
12. Viola, P., Ralli, M., Pisani, D., Malanga, D., Sculco, D., Messina, L., Laria, C., Aragona, T., Leopardi, G., Ursini, F., Scarpa, A., Topazio, D., Cama, A., Vespertini, V., Quintieri, F., Cosco, L., Cunsolo, E. M., Chiarella, G. (2020). Tinnitus and equilibrium disorders in COVID-19 patients: preliminary results. EUROPEAN ARCHIVES OF OTO-RHINO-LARYNGOLOGY. DOI 10.1007/s00405-020-06440-7. PM 33095432.
13. Anzivino, R., Sciancalepore, P. I., Petrone, P., D'Elia, A., Petrone, D., Quaranta, N. Tinnitus revival during COVID-19 lockdown: how to deal with it? (2021). EUROPEAN ARCHIVES OF OTO-RHINO-LARYNGOLOGY. DOI 10.1007/s00405-020-06147-9. PM 32572563.
14. Little, C., Cosetti, M. K. (2020). A Narrative Review of Pharmacologic Treatments for COVID-19: Safety Considerations and Ototoxicity. LARYNGOSCOPE. DOI 10.1002/lary.29424. PM 33491234.
15. Maharaj, S., Alvarez, M. B., Mungul, S., Hari, K. (2020). Otologic dysfunction in patients with COVID-19: A systematic review. LARYNGOSCOPE INVESTIGATIVE OTOLARYNGOLOGY. DOI 10.1002/lio2.498. PM 33365394.
16. Beukes, E. W., Baguley, D. M., Jacquemin, L., Lourenco, M. P. C. G., Allen, P. M., Onozuka, J., Stockdale, D., Kaldo, V., Andersson, G., Manchaiah, V. (2020). Changes in Tinnitus Experiences During the COVID-19 Pandemic. FRONTIERS IN PUBLIC HEALTH. DOI 10.3389/fpubh.2020.592878. PM 33251179.
17. Naylor, G., Burke, L. A., Holman, J. A. (2020). Covid-19 Lockdown Affects Hearing Disability and Handicap in Diverse Ways: A Rapid Online Survey Study. EAR AND HEARING. DOI 10.1097/AUD.0000000000000948. PM 33136621.
18. Micarelli, A., Granito, I., Carlino, P., Micarelli, B., Alessandrini, M. (2020). Self-perceived general and ear-nose-throat symptoms related to the COVID-19 outbreak: a survey study during quarantine in Italy. JOURNAL OF INTERNATIONAL MEDICAL RESEARCH. DOI 10.1177/0300060520961276. PM 33081538.

19. Saunders, G. H. & Roughley, A. (2020). Audiology in the time of COVID-19: practices and opinions of audiologists in the UK. INTERNATIONAL JOURNAL OF AUDIOLOGY. DOI 10.1080/14992027.2020.1814432. PM 32909474.
20. Schlee, W., Holleland, S., Bulla, J., Simoes, J., Neff, P., Schoisswohl, S., Woelflick, S., Schecklmann, M., Schiller, A., Staudinger, S., Probst, T., Langguth, B. (2020). The Effect of Environmental Stressors on Tinnitus: A Prospective Longitudinal Study on the Impact of the COVID-19 Pandemic.JOURNAL OF CLINICAL MEDICINE. DOI 10.3390/jcm9092756. PM 32858835.
21. Almufarrij, I. & Munro, K. J. (2021). One year on: an updated systematic review of SARS-CoV-2, COVID-19 and audio-vestibular symptoms. INTERNATIONAL JOURNAL OF AUDIOLOGY. DOI 10.1080/14992027.2021.1896793. PM 3375025.
22. Xia, L., He, G., Feng, Y., Yu, X. X., Zhao, X. L., Yin, S. K., Chen, Z. N., Wang, J., Fan, J. G., Dong, C. (2021). COVID-19 associated anxiety enhances tinnitus. PLOS ONE. DOI 10.1371/journal.pone.0246328. PM 33544744.
23. Vijayasundaram, S., Karthikeyan, P., Mehta, S. D. (2020). Proficiency of virtual follow-up amongst tinnitus patients who underwent intratympanic steroid therapy amidst COVID 19 pandemic. AMERICAN JOURNAL OF OTOLARYNGOLOGY. DOI 10.1016/j.amjoto.2020.102680. PM 32861124.
24. Saniasiaya, J. & Kulasegarah, J. (2020). Auditory Cinchonism in COVID. ENT-EAR NOSE & THROAT JOURNAL. DOI 10.1177/0145561320947255. PM 32744901.
25. Aazh, H., Swanepoel, D., Moore, B. C. J. (2020). Telehealth tinnitus therapy during the COVID-19 outbreak in the UK: uptake and related factors. INTERNATIONAL JOURNAL OF AUDIOLOGY. DOI 10.1080/14992027.2020.1822553. PM 33000663.

Table S3.
Quality assessment ratings

Table S3a. The overall quality rating for case reports and case series studies using the National Institutes of Health's Quality Assessment Tool for Case Series.

	Q1	Q2	Q3	Q4	Q5	Q6	Q7	Q8	Q9	Overall quality rating		
										Reviewer 1	Reviewer 2	Final rating
Chirakkal et al. (2020)	Y	Y	NA	NA	Y	Y	CD	NA	Y	Fair	Fair	Fair
Cui et al. (2020)	Y	Y	CD	CD	NA	CD	CD	NA	N	Fair	Fair	Fair
Degan et al. (2020)	Y	Y	NA	NA	Y	CD	NA	NA	Y	Fair	Fair	Fair
Fidan (2020)	Y	Y	NA	NA	Y	Y	Y	NA	Y	Fair	Fair	Fair
Karimi-Galougahi et al. (2020)	N	Y	N	Y	NA	Y	CD	NA	Y	Fair	Fair	Fair
Koumpa et al. (2020)	Y	Y	NA	NA	Y	Y	CD	NA	Y	Good	Good	Good
Lamounier et al. (2020)	Y	Y	NA	NA	Y	Y	NA	NA	Y	Good	Good	Good
Lang et al. (2020)	N	Y	NA	NA	Y	Y	CD	NA	Y	Fair	Fair	Fair
Maharaj and Hari. (2020)	Y	Y	NA	NA	Y	Y	CD	NA	Y	Fair	Good	Good
Rhman and Wahid (2020)	N	Y	NA	NA	Y	Y	Y	NA	Y	Fair	Fair	Fair
Sun et al. (2020)	N	Y	NA	NA	NA	N	CD	NA	N	Poor	Poor	Poor

Y = Yes, N = No, NR = not reported; NA,= not applicable; CD = cannot determine.
Q1 = Was the study question or objective clearly stated? Q2 = Was the study population clearly and fully described, including a case definition? Q3 = Were the cases consecutive? Q4 = Were the subjects comparable? Q5* = Was the intervention clearly described? (*Due to nature of the review and our study aims, we adapted question 5 on NIH quality assessment for case reports to exposure instead of intervention) Q6 = Were the outcome measures clearly defined, valid, reliable, and implemented consistently across all study participants? Q7 = Was the length of follow-up adequate? Q8 = Were the statistical methods well-described? Q9 = Were the results well-described?

Table S3b. The overall quality rating for observational and cross-sectional studies using the National Institutes of Health's Quality Assessment Tool for Observational Cohort and Cross-Sectional Studies.

Reference	Q1	Q2	Q3	Q4	Q5	Q6	Q7	Q8	Q9	Q10	Q11	Q12	Q13	Q14	Overall quality rating		
															Reviewer 1	Reviewer 2	Final rating
Beukes et al. (2020)	Y	Y	Y	Y	N	Y	CD	CD	CD	N	Y	NA	NA	CD	Fair	Fair	Fair
Daikhes et al. (2020)	Y	N	N	N	N	Y	CD	CD	CD	CD	N	NA	CD	N	Poor	Poor	Poor
Davis et al. (2020)	Y	Y	CD	Y	N	Y	CD	CD	CD	CD	N	Y	CD	N	Fair	Fair	Fair
Elibol (2020)	Y	Y	CD	Y	N	Y	CD	CD	CD	N	N	NA	CD	CD	Fair	Fair	Fair
Freni et al. (2020)	Y	Y	CD	Y	N	Y	CD	CD	CD	Y	Y	NA	CD	CD	Good	Fair	Fair
Iqbal et al. (2021)	Y	Y	CD	Y	Y	N	CD	CD	CD	N	Y	NA	NA	Y	Fair	Good	Good
Kamal et al. (2020)	Y	Y	CD	CD	N	N	CD	CD	CD	N	Y	NA	NA	Y	Fair	Fair	Fair
Klopfenstein et al., (2020)	Y	Y	CD	Y	N	Y	CD	CD	CD	CD	N	NA	NA	CD	Fair	Fair	Fair
Korkmaz et al. (2020)	Y	Y	Y	Y	N	Y	CD	CD	CD	N	Y	NA	NA	Y	Fair	Fair	Fair
Lechien et al. (2020)	Y	Y	CD	CD	N	Y	CD	CD	CD	N	Y	CD	NA	N	Fair	Fair	Fair
Liang et al. (2020)	Y	Y	CD	Y	N	Y	CD	CD	CD	CD	N	NA	CD	CD	Fair	Fair	Fair
Micarelli et al. (2020)	Y	Y	CD	Y	N	N	CD	CD	CD	NR	N	Y	CD	N	Fair	Fair	Fair
Munro et al. (2020)	Y	N	Y	Y	N	Y	CD	CD	CD	CD	N	N	Y	CD	Fair	Fair	Fair
Naylor et al. (2020)	Y	Y	Y	Y	N	N	CD	CD	CD	N	Y	NA	NA	Y	Fair	Fair	Fair
Savtale et al (2021)	Y	Y	CD	Y	N	Y	CD	CD	CD	CD	N	N	Y	N	Fair	Fair	Fair
Swain & Pani (2021)	Y	Y	Y	Y	N	Y	CD	CD	CD	N	Y	NA	NA	N	Fair	Fair	Fair
Viola et al. (2020)	Y	Y	Y	Y	N	Y	CD	CD	CD	CD	N	Y	NA	N	Fair	Fair	Fair
Zayet et al. (2020)	Y	Y	Y	Y	N	Y	CD	CD	CD	N	Y	NA	NA	Y	Fair	Fair	Fair

Q = Question: Y=yes; N = no; NR = not reported; NA = not applicable; CD = cannot determine.
Q1 = Was the research question or objective in this paper clearly stated? Q2 = Was the study population clearly specified and defined? Q3 = Was the participation rate of eligible persons at least 50%? Q4 = Were all the subjects selected or recruited from the same or similar populations (including the same time period)? Were inclusion and exclusion criteria for being in the study prespecified and applied uniformly to all participants? Q5 = Was a sample size justification, power description, or variance and effect estimates provided? Q6 = For the analyses in this paper, were the exposure(s) of interest measured prior to the outcome(s) being measured? Q7 = Was the timeframe sufficient so that one could reasonably expect to see an association between exposure and outcome if it existed? Q8 = For exposures that can vary in amount or level, did the study examine different levels of the exposure as related to the outcome (e.g., categories of exposure, or exposure measured as continuous variable)? Q9 = Were the exposure measures (independent variables) clearly defined, valid, reliable, and implemented consistently across all study participants? Q10 = Was the exposure(s) assessed more than once over time? Q11 = Were the outcome measures (dependent variables) clearly defined, valid, reliable, and implemented consistently across all study participants? Q12 = Were the outcome assessors blinded to the exposure status of participants? Q13 = Was loss to follow-up after baseline 20% or less? Q14 = Were key potential confounding variables measured and adjusted statistically for their impact on the relationship between exposure(s) and outcome(s)?

Table S3c. Quality assessment for pre-post studies using the National Institutes of Health's Quality Assessment Tool for Before-After (Pre-Post) Studies with No Control Group

Reference	Q1	Q2	Q3	Q4	Q5	Q6	Q7	Q8	Q9	Q10	Q11	Q12	Overall quality rating		
													Reviewer 1	Reviewer 2	Final rating
Anzivino et al. (2020)	Y	N	Y	Y	N	Y	Y	NA	CD	N	N	NA	Poor	Poor	Poor
Schlee et al. (2020)	Y	Y	Y	Y	Y	Y	Y	NA	N	Y	N	Y	Fair	Fair	Fair
Xia et al. (2020)	Y	Y	Y	Y	Y	Y	Y	NA	Y	Y	N	Y	Fair	Fair	Fair

Q = Question: Y=yes; N = no; NR = not reported; NA = not applicable; CD = cannot determine.
Q1 = Was the study question or objective clearly stated? Q2 = Were eligibility/selection criteria for the study population prespecified and clearly described? Q3 = Were the participants in the study representative of those who would be eligible for the test/service/intervention in the general or clinical population of interest? Q4 = Were all eligible participants that met the prespecified entry criteria enrolled? Q5 = Was the sample size sufficiently large to provide confidence in the findings? Q6 = Was the test/service/intervention clearly described and delivered consistently across the study population? Q7 = Were the outcome measures prespecified, clearly defined, valid, reliable, and assessed consistently across all study participants? Q8 = Were the people assessing the outcomes blinded to the participants' exposures/interventions? Q9 = Was the loss to follow-up after baseline 20% or less? Were those lost to follow-up accounted for in the analysis? Q10. Did the statistical methods examine changes in outcome measures from before to after the intervention? Were statistical tests done that provided p values for the pre-to-post changes? Q11. Were outcome measures of interest taken multiple times before the intervention and multiple times after the intervention (i.e., did they use an interrupted time-series design)? Q12. If the intervention was conducted at a group level (e.g., a whole hospital, a community, etc.) did the statistical analysis take into account the use of individual-level data to determine effects at the group level?
